# Losartan and Eprosartan Induce a Similar Effect on the Acute Rise in Serum Uric Acid Concentration after an Oral Fructose Load in Patients with Metabolic Syndrome

**DOI:** 10.1155/2021/2214978

**Published:** 2021-08-25

**Authors:** Anna Masajtis-Zagajewska, Jacek Majer, Michał Nowicki

**Affiliations:** Department of Nephrology, Hypertension and Kidney Transplantation, Medical University of Lodz, Central University Hospital, Lodz, Poland

## Abstract

**Introduction:**

Excessive intake of fructose increases serum uric acid concentration. Hyperuricemia induces a negative effect on atherosclerosis and inflammation. Hyperuricemia is common in patients with arterial hypertension. Several antihypertensive drugs including diuretics increase serum uric acid concentration. In contrast, the angiotensin II receptor antagonist (ARB) losartan was found to lower serum uric acid though it may increase renal excretion while other ARBs showed mostly a neutral effect. In this study, effects of two AT1 receptor antagonists losartan and eprosartan on serum uric acid changes induced by oral fructose load were directly compared.

**Methods:**

The randomized, crossover, head-to-head comparative study comprised 16 ambulatory patients (mean age 64.5 ± 9.8 years). The patients fulfilled AHA/NHLBI 2005 criteria of metabolic syndrome. A daily single morning dose of each study drug (50 mg of losartan or 600 mg of eprosartan) was given during two 3-month periods in a random order separated by 2-week washout time. The oral fructose tolerance test (OFTT) was performed at baseline and after each two 3-onth treatment periods. Before and during OFTT, urine excretion of uric acid and creatinine was assessed in the first morning portion of urine. Blood samples for the measurement of serum uric acid and lipids were taken at baseline and 30, 60, and 120 minutes after oral intake of 75 g of fructose.

**Results:**

After 3-month treatment with eprosartan and losartan, both systolic and diastolic blood pressure decreased significantly and to a similar extent. After the treatment, serum uric acid and its baseline and postfructose urine excretion were unchanged. No significant changes of plasma lipids before and after OFTT were observed throughout the study.

**Conclusions:**

The study showed that in patients with hypertension and metabolic syndrome, both losartan and eprosartan have a neutral effect on fasting and postfructose load serum uric acid concentration and its urinary excretion. This trial is registered with NCT04954560.

## 1. Introduction

Hyperuricemia is seen in about 20% of adults in the general population [1] and is mostly a consequence of abnormal purine metabolism caused either by an excessive uric acid production or by its impaired elimination by the kidneys [2].

On the one hand, high uric acid concentration may be an important link in the pathogenesis of hypertension, but on the other hand, in mild and moderate hypertension, uric acid excretion is frequently impaired [3]. A study showed that in the hypertensive patients with hyperuricemia, renal blood flow is reduced compared to that in the patients with arterial hypertension but with normal uric acid concentration [4].

Several studies showed that the prevalence of hyperuricemia in patients with hypertension is much higher than that in the general population and may worsen after the onset of antihypertensive treatment [5]. That may indicate that hyperuricemia may be also caused by antihypertensive drugs. In contrast to diuretics and nonselective beta blockers, the agents that block the renin-angiotensin-aldosterone system have had a neutral effect on serum uric acid [5–7]. Several clinical studies showed that losartan in contrast to other AT1 receptor agonists (ARB) may have specific uricosuric properties and thereby can lower uric acid concentration [8–11]. It is not clear whether the effect is clinically relevant and may add to organ protective effects of the drug. It has been speculated that the uricosuric effect could make losartan particularly useful for the treatment of arterial hypertension associated with hyperuricemia and metabolic syndrome [9, 10].

The uricosuric effect of losartan is most likely due to overlapping two different mechanisms regulating the excretion of uric acid. Losartan may increase uric acid tubular secretion in the same way as other inhibitors of the renin-angiotensin-aldosterone system, but in addition, it may specifically inhibit postsecretory resorption of uric acid in the proximal tubule. The effect may be due to a specific structure of the losartan molecule [11]. The urate-anion transporter is a monoammonium-selective transporter, and the losartan molecule is mainly a monoanion at a normal pH range (as opposed to dianion, e.g., eprosartan) and therefore is a good substrate for the exchanger [12]. However, this concept remains speculative since, e.g., irbesartan which is also a monoanion has no consistent uricosuric effect [13].

An increased accessibility of the high-calorie food contributes to the worldwide epidemic of the metabolic syndrome. The rapidly increasing rate of obesity and metabolic syndrome may be partly related to an excessive consumption of fructose added to sweetened beverages and food [14–16]. Fructose, in contrast to other carbohydrates, causes an increase in serum uric acid concentration, which may facilitate the development of the metabolic syndrome [17–19].

The aim of our study was to directly compare the effect of two AT1 receptor antagonists, losartan and eprosartan, on serum uric acid changes stimulated by an oral fructose load.

## 2. Material and Methods

The study group included 16 patients (15 F, 1 M, mean age 64.5 ± 9.8 years). The patients selected for the study fulfilled the AHA/NHLBI 2005 criteria of the metabolic syndrome [19] and ESC/ESH criteria of arterial hypertension. The exclusion criteria included an antihypertensive therapy with the renin-angiotensin-aldosterone axis blocking agent used anytime during the last 3 months; current or past therapy with the SGLT2 inhibitor, GLP-1 agonist, or DPP4-inhibitor; suspected or confirmed secondary form of hypertension; estimated glomerular filtration rate < 60 ml/min/1.73 m^2^; chronic liver disease; acute infection; psychiatric disorders; mean serum potassium concentration at the last three measurements < 4.0 mmol/l, or aspartate aminotransferase or alanine aminotransferase or creatinine kinase > ×1.5 upper range limit. Seven patients had diabetes mellitus of which 4 were treated with insulin. Twelve patients were receiving metformin. Waist circumference was measured on the initial visit. Plasma lipids and blood glucose were measured in a fasting state during the study.

The study was designed as a randomized, crossover, head-to-head comparative study. Randomization was carried out using an MS Excel random number generator. After qualification, each patient was randomly assigned to receive either losartan (Lorista, KRKA, Slovenia) or eprosartan (Teveten, Solvay Pharmaceuticals, Australia). The patients were taking all other previously prescribed drugs in unmodified doses during the whole course of the study. Each study drug was given in a random order as a single morning dose (50 mg of losartan or 600 mg of eprosartan) for two periods each lasting 3 months separated by 2-week washout time.

Losartan (C_22_H_23_CIN_6_O) is approximately 33% orally bioavailable and has a *T*_max_ of 1 hour, and its active metabolite has a *T*_max_ of 3-4 hours. The terminal elimination half-life of losartan is 1.5-2.5 hours while the active metabolite has a half-life of 6-9 hours [20].

Eprosartan (C_23_H_24_N_2_O_4_S) has an absolute bioavailability after a single 300 mg oral dose of 13%. Food delays absorption of the drug. Eprosartan is not metabolized by the cytochrome P450 system and is eliminated as unchanged drug. Less than 2% of an oral dose is excreted in the urine. The terminal elimination half-life of eprosartan following oral administration is 5 to 9 hours [21].

Oral fructose tolerance with the administration of 75 g of fructose was conducted 3 times during the study in each patient, i.e., at baseline and after each of the treatment periods. Before the commencement of OFTT, the patients collected the urine for 2 hours for assessment of the urinary excretion of uric acid and creatinine. Other baseline measurements included blood pressure, serum concentration of glucose, uric acid, creatinine and plasma lipids (total, HDL, and LDL cholesterol and triglycerides). Subsequent blood samples were taken three times during each OFTT, i.e., after 30, 60, and 120 minutes from its start to determine serum uric acid concentration. In addition, peripheral blood pressure was measured before the collection of each blood sample. After 120 minutes, blood was also taken to assess plasma lipids. The second timed 2-hour urine collection was obtained during OFTT. The same procedures were repeated after each treatment period. The patients took all their prescribed medication including the study drug in the morning 120 minutes before the beginning of OFTT.

Routine automated laboratory tests were used to assess blood and urine parameters. Blood pressure was taken in a sitting position with the aneroid sphygmomanometer. Blood pressure was measured both at baseline and after 3-month therapy with each study drug. The mean from four measurements obtained between 0 and 120 minutes of OFTT was taken for the analysis. Blood pressure was measured by a designated single member of the staff. Mean blood pressure (MAP) was calculated for all measurements as diastolic BP + 1/3 of pulse pressure (systolic BP–diastolic BP).

The results are expressed as mean ± SD or median with interquartile range depending on each variable distribution. 95% confidence intervals were calculated for the changes of the parameters caused by the treatment. Statistical significance was defined at *p* < 0.05. The within-group comparisons were analyzed using one-way ANOVA and *t*-test for normally distributed variables or alternatively with the nonparametric Wilcoxon test. The normality of the variable distribution was checked with the Shapiro-Wilk test. The Pearson or Spearman correlation coefficient was used to assess the relations between variables depending on their distribution. The area under the curve (AUC) of serum uric acid during oral fructose tolerance test was calculated using the trapezoidal rule.

## 3. Results

Twenty-two patients were recruited to the study; after screening, 6 patients did not complete the study procedure and therefore were not included in the final per-protocol data analysis; in total, 16 patients were included. All dropouts were due to gastrointestinal intolerance of oral fructose. No side effects of the study drugs were observed. Baseline clinical characteristics of the study population are presented in Table 1.

Table 2 shows the AHA/NHLBI 2005 criteria for metabolic syndrome and the pass criteria by patients in the study group.

Systolic, diastolic, and mean blood pressure decreased significantly and to a similar extent from baseline after 3 months of eprosartan and losartan administration.  Table 3 shows systolic, diastolic, and mean blood pressure values during the study.

No significant changes of blood glucose and serum creatinine were observed throughout the study.  Table 4 shows the effect of 3-month treatment with losartan or eprosartan on serum concentration of uric acid and urine uric acid excretion. Serum uric acid concentration and urine uric acid excretion did not significantly change after losartan nor after eprosartan administration.

**Figure 1:**
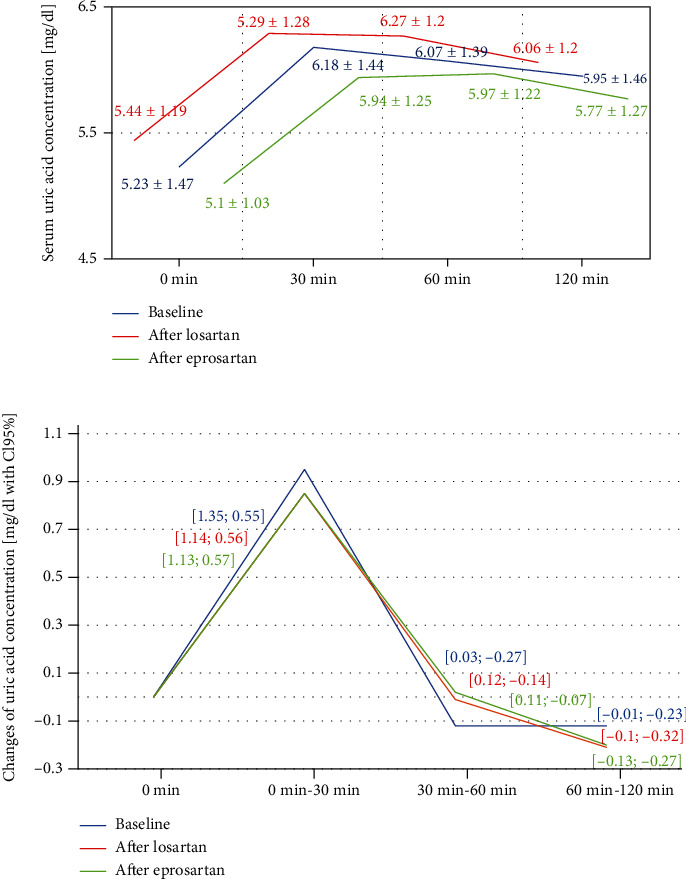
Serum uric acid concentration during OFTT (a) and changes of serum uric acid concentration during OFTT (b).

**Figure 2:**
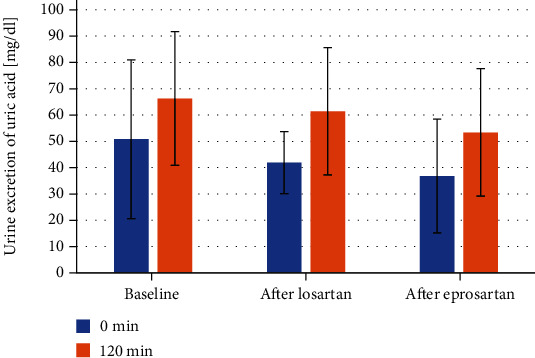
Urine excretion of uric acid during OFTT.

Serum uric acid increased significantly during OFTT after the therapy with either losartan or eprosartan (*p* < 0.001 in both study periods).  Figure 1 shows the values of serum uric acid concentration on baseline and after 30, 60, and 120 minutes after OFTT and the changes of serum uric acid from baseline after 30, 60, and 120 minutes after ingestion of an acute oral fructose load. The changes of serum acid caused by oral fructose load during OFTT were comparable after both treatment periods. Urinary uric acid excretion after oral fructose load was not changed after treatment with losartan or eprosartan (Figure 2). AUC of serum uric acid concentration during OFTT after treatment with eprosartan (11.6 ± 2.4) and losartan (12.2 ± 2.4) and at baseline (11.9 ± 2.8) was similar.

## 4. Discussion

The main finding of our study was that the angiotensin receptor blocker, losartan—which is believed to lower UA levels—was associated with similar serum uric acid levels to those of a chemically distinct angiotensin receptor antagonist eprosartan. This lack of difference occurred despite the other ARB not attaching to the same renal receptor to which the losartan molecule attaches and supposedly lowers UA serum levels. From these results, we conclude that losartan did not significantly affect serum concentration and urinary excretion of uric acid, as well as the magnitude of the changes of serum uric acid concentration after the oral fructose load in comparison to other ARBs.

Our results contrasting with the findings of several previous studies have not confirmed a class effect of angiotensin receptor blockers (ARB) on serum uric acid [22–25]. While most currently available ARBs appear to have a neutral effect on uricemia, losartan was found to specifically increase the urinary excretion of uric acid and thereby caused a significant reduction of serum uric acid [13, 24–27].

Although some research demonstrated an increased urinary uric acid excretion and a decrease in serum uric acid during losartan therapy, there is only a scarce evidence from head-to-head studies in which the effect of the drug was compared to that of other ARBs in the patients with arterial hypertension [24, 27, 28]. The results of these studies were not concordant. In two small studies [24, 28], losartan significantly increased urinary uric acid excretion, but only in one of them, a simultaneous decrease in serum uric acid was observed [28]. Another randomized study [29] carried out in a group of hypertensive patients after kidney transplantation treated with a cyclosporin-based triple immunosuppressive regimen showed only a small and nonsignificant decrease in serum uric acid after losartan.

Unlike our study, all previous studies investigated only the effect of losartan on a fasting serum level of uric acid but did not assess the effect of this drug on the changes of serum uric acid induced by dietary stimuli. That was also the case in the study [24] which compared the effect of 50 mg daily losartan and 600 mg eprosartan on uric acid metabolism in patients with mild to moderate hypertension. In this study, no significant changes of serum uric acid were reported despite a significant increase in urinary excretion of uric acid seen in the losartan group. The authors hypothesized that a treatment period of only 4 weeks might be a reason for a lack of any significant effect. An additional difficulty in the interpretation of the results was that a low purine diet was observed for only one day before urine was collected.

Our study was designed to address the uncertainty around the effects of different ARBs on uricemia. The crossover design of the study allowed a direct comparison of the effect of both drugs in the same subject and in the same condition [30]. It is of note that several patients, who were qualified to the study, took a small dose of aspirin (75 mg), *β*-blocker, or diuretic which may have a weak uricosuric activity [7, 31, 32]. These drugs were however administered in an unchanged dose during the whole study and therefore did not have the effect on the comparison between two study drugs.

Schmidt et al. [28] showed that the uricosuric effect of losartan appears to wane over time. In addition, the majority of our patients had only mildly elevated serum uric acid level at baseline which could also limit a potential magnitude of the effect of ARB on its metabolism. The baseline uric acid level in most previous studies was higher than that in our study [23, 25, 28].

Three months of treatment with eprosartan or losartan led to an expected significant decrease in arterial pressure. It should be noted that our patients had a well-controlled blood pressure already at the beginning of the study, so the addition of further antihypertensive drug only had a small effect on blood pressure.

## 5. Conclusion

Our study demonstrated that losartan did not specifically affect serum concentration and urinary excretion of uric acid, as well as the magnitude of the changes of serum uric acid concentration after the oral fructose load. Therefore, we were not able to confirm the usefulness of losartan compared to other ARBs in the treatment of arterial hypertension in the patients with metabolic syndrome.

## Figures and Tables

**Table 1:** Baseline clinical and biochemical characteristics of the study group.

	Mean ± SD
Age (years)	64.5 ± 9.8
Body mass index (kg/m^2^)	33.7 ± 4.6
Uric acid (mg/dl)	5.23 ± 1.4
Uric acid excretion (mg/dl)	50.8 ± 30.2
Total cholesterol (mg/dl)	218.4 ± 42.5
HDL cholesterol (mg/dl)	43.7 ± 13.2
LDL cholesterol (mg/dl)	138.7 ± 26.9
Triglycerides (mg/dl)	185.1 ± 131.3
Creatinine (mg/dl)	1.0 ± 0.2
Glucose (mg/dl)	131.3 ± 74
Systolic blood pressure (mmHg)	139.7 ± 17.6
Diastolic blood pressure (mmHg)	82.8 ± 8.4
Mean arterial pressure (mmHg)	100.1 ± 13.8
Antihypertensive therapy	
Ca antagonist	3 (19%)
Loop diuretic	0 (0%)
Thiazide diuretic	5 (31%)
*β*-Blocker	6 (37%)
*α*-Blocker	1 (6%)
Antidiabetic therapy	
Insulin	4 (25%)
Metformin	12 (74%)
Sulfonylurea	1 (6%)

**Table 2:** The AHA/NHLBI 2005 criteria for metabolic syndrome [19] and the pass criteria by patients in the study group.

Patient	Sex	HDL (mg/dl)	Triglyceride (mg/dl)	Waist circumference (cm)	Hypertension or pharmacologic therapy for hypertension	Diabetes mellitus or receiving pharmacologic therapy for elevated fasting glucose levels	Hypolipidemic therapy	Number of metabolic syndrome criteria met
1	F	27.5	329.9	116	Y	Y	Y	5
2	F	53	152	120	Y	Y	N	4
3	F	52.7	281.7	118	Y	Y	N	4
4	F	45	142	112	Y	N	Y	4
5	F	59.7	257	106	Y	N	Y	4
6	F	67	119	111	Y	N	Y	3
7	F	56.3	153.2	92	Y	N	Y	4
8	F	63.2	140.8	114	Y	Y	Y	4
9	F	60	170	124	Y	N	N	3
10	F	44	211	106	Y	Y	N	5
11	M	85.8	129	103	Y	N	Y	3
12	F	53.5	139	90	Y	Y	Y	4
13	F	36	315	90	N	N	Y	4
14	F	41.9	144	111	Y	Y	N	5
15	F	34	442	116	Y	Y	N	5
16	F	46.7	134.3	106	Y	Y	Y	5

The AHA and NHLBI require at least three of the following criteria for the diagnosis of the metabolic syndrome: (i) waist circumference of at least 40 inches (102 cm) in men or 35 inches (89 cm) in women, measured at the top of the iliac crest at the end of a normal expiration; (ii) triglyceride level of at least 150 mg per dl (1.70 mmol per l) or receiving pharmacologic therapy for elevated triglyceride levels; (iii) HDL cholesterol level of less than 40 mg per dl (1.05 mmol per L) in men or less than 50 mg per dl (1.30 mmol per L) in women or receiving pharmacologic therapy for reduced HDL cholesterol levels; (iv) systolic blood pressure of at least 130 mmHg or diastolic blood pressure of at least 85 mmHg or receiving pharmacologic therapy for hypertension; (v) fasting glucose level of at least 100 mg per dl (5.6 mmol per L) or receiving pharmacologic therapy for elevated fasting glucose levels.

**Table 3:** Systolic blood pressure, diastolic blood pressure, and mean arterial pressure at baseline and after 12-week treatment with losartan or eprosartan.

	Baseline	After losartan	*p* value	After eprosartan	*p* value
Systolic BP (mmHg)	139.7 ± 17.6	129.3 ± 24.0	0.04	128.7 ± 12.2	0.01
Diastolic BP (mmHg)	82.8 ± 8.4	75.9 ± 10.4	0.03	79.7 ± 9.4	0.04
MAP (mmHg)	100.1 ± 13.8	93.8 ± 13.7	0.04	95.9 ± 9.1	0.03

BP: blood pressure; MAP: mean arterial pressure.

**Table 4:** Serum concentration of uric acid and uric acid excretion before and after 12-week treatment with losartan or eprosartan.

	Baseline	After losartan	*p* value	After eprosartan	*p* value
Uric acid (mg/dl)	5.23 ± 1.4	5.44 ± 1.19	0.462	5.1 ± 1.03	0.528
Uric acid urinary excretion (mg/dl)	50.8 ± 30.2	41.9 ± 21.6	0.34	36.8 ± 11.8	0.08

## Data Availability

The data presented in this study are available on request from the corresponding author.
